# Prevalence of hypospadias in newborns in tropical province of China: A cross-sectional population-based study, 2014–2023

**DOI:** 10.1097/MD.0000000000042307

**Published:** 2025-05-09

**Authors:** Weizhen Bu, Xiaohua Li, Xiaojing Hu, Yan Xuan, Zhenli Zhao

**Affiliations:** a Department of Pediatric Urology, Hainan Women and Children’s Medical Center, Haikou, China; b Department of Urology, Hainan Women and Children’s Medical Center, Haikou, China; c Department of Nursing, Hainan Women and Children’s Medical Center, Haikou, China.

**Keywords:** birth defects, epidemiology, hypospadias, prevalence

## Abstract

Hypospadias is among the most prevalent urogenital malformations in newborns exhibiting significant global variations in prevalence. However, there is a notable scarcity of epidemiological data regarding this condition in Hainan. The aim of the current study was to determine and analyze the prevalence of hypospadias in the tropical province over the past decade. Data from the China Maternal and Child Health Surveillance System of Hainan (2014–2023) were utilized to calculate the prevalence rates of hypospadias, stratified by birth year, maternal age, and maternal residence. During the study period, a total of 304 cases of hypospadias were identified among 549,833 births, resulting in a prevalence rate of 5.53 per 10,000 births over the past 10 years in Hainan. The overall prevalence increased from 3.15 to 8.36 per 10,000 births. Significant variations were observed across different maternal age groups: less than 20 years (5.12 per 10,000), 20 to 24 years (3.84 per 10,000), 25 to 29 years (5.31 per 10,000), 30 to 34 years (7.00 per 10,000), and 35 years or older (5.68/per 10,000). However, no statistically significant differences were found in the prevalence of hypospadias based on maternal residence, with urban areas showing a prevalence of 6.08 per 10,000 compared with 4.84 per 10,000 in rural areas. Our data indicate that the prevalence of hypospadias was 5.53 per 10,000 from 2014 to 2023, exhibiting distinct temporal and age distribution characteristics. Therefore, understanding and monitoring the prevalence of hypospadias remains essential. It is crucial to enhance health education regarding birth health counseling and to promote regular perinatal examination to reduce the incidence of hypospadias.

## 1. Introduction

Hypospadias, one of the most prevalent anomalies of the human genitourinary system, is characterized by a displacement of the urethral opening along the penile shaft that, affecting approximately 1 in every 125 live-born male infants.^[1]^ Although surgical correction of the urogenital structure is possible, individuals with hypospadias frequently experience ongoing functional difficulties,^[2]^ psychosocial and psychosexual dysfunction,^[3–7]^ surgical complications,^[8,9]^ and co-occurring health conditions that extend beyond birth defects.^[10,11]^ While several studies have reported on the epidemiology of hypospadias, its prevalence varies across different regions and countries.^[12–14]^ Therefore, this study aims to analyze the prevalence patterns and trends of hypospadias in Hainan from 2014 to 2023, utilizing data from the China Maternal and Child Health Surveillance System.

## 2. Materials and methods

Population data spanning from 2014 to 2023 were sourced from the China Maternal and Child Health Surveillance System. This dataset included the number of birth defects alongside perinatal and maternal general characteristics. Each birth defect case form documented demographic information, birth date, sex, gravidity, parity, gestational age, birth weight, outcomes, and any birth defects diagnosed within the first even days postdelivery. External birth defects were diagnosed through physical examinations, while visceral anomalies were primarily identified using ultrasonography or X-ray examinations. Chromosomal or genetic diseases were diagnosed via molecular diagnostic methods. Birth defects were coded by trained professionals according to the International Classification of Diseases (10th edition). At each site, trained surveillance staff at the county/district and village/community levels were responsible for routine quarterly data collection, verification, follow-up, and online reporting. These procedures adhered to the technical guide of the China Maternal and Child Health Monitoring Surveillance System, ensuring the accuracy and reliability of the surveillance data. The study complied with the World Medical Association Declaration of Helsinki regarding ethical conduct in research and received approval from the Ethics Committee of Hainan Women and Children’s Medical Center.

### 2.1. Statistical analysis

The prevalence rate of hypospadias was defined as the number of cases per 10,000 births. Statistical analyses were conducted using Statistical Package for the Social Sciences (SPSS) version 24.0. The trend in prevalence rates was estimated using the chi-square trend test. Differences between categorical data were assessed using either the chi-square test or Fisher’s exact test. A *P*-value of less than .05 was considered statistically significant.

## 3. Results

Table 1 presents the fundamental characteristics and perinatal outcomes of hypospadias cases in the current study. Between 2014 and 2023, a total of 304 hypospadias cases were identified among 549,833 pregnant women who participated in prenatal screening within the study cohort. Among the cases, 144 infants weighed less than 2500 g, while 160 weighed 2500 g or more. In addition, 108 cases were delivered before 37 weeks of gestation, whereas 196 cases were delivered at or after 37 weeks. The majority of hypospadias cases were singletons (90.13%), and approximately 42.44% of the cases were born to primiparous women. Furthermore, the perinatal mortality rate among hypospadias cases was recorded at 1.32%.

**Table 1:** Characteristics of hypospadias cases in newborns.

Characteristics	Frequency (%)
Birth weight	
<2500 g	144 (47.37)
≥2500 g	160 (52.63)
Gestational weeks	
<37 week	108 (35.53)
≥37 week	196 (64.47)
Perinatal outcome	
Stillbirth	4 (1.32)
Live birth	300 (98.68)
Nationality	
Han	272 (89.47)
Minorities	32 (10.53)
Singleton	
Yes	274 (90.13)
No	30 (9.86)
Parity	
1	175 (57.56)
2	108 (35.53)
≥3	21 (6.91)
Sexual differentiation	
Yes	4 (1.32)
No	300 (98.68)
Congenital heart disease	
Yes	20 (6.58)
No	284 (93.42)
Other malformations	
Yes	21 (6.90)
No	287 (93.10)
Estrogenic or endocrine-disrupting antiandrogen agents	
Yes	7 (2.30)
No	297 (97.70)

Table 2 presents the prevalence rate of hypospadias stratified by maternal residence and maternal age. The prevalence was higher in urban areas compared with rural, areas; however, this difference was not statistically significant. Among the five levels of maternal age categories, the chi-square test confirmed significant variations in prevalence. Further refinement of the analysis reduced the differences between maternal age groups, with only the 30 to 34 years group showing a significant effect on hypospadias prevalence (Table 2).

**Table 2:** Prevalence rates of hypospadias stratified by birth year, maternal residence, and maternal age (per 10,000 births).

Characteristics	Total birth	Case (%)	*P* value
Maternal residence			
Urban	304,042	185 (6.08)	.051
Rural	245,791	119 (4.84)	
Maternal age			
<20	25,402	13 (5.12)	.021
20	106,638	41 (3.84)	
25	188,285	100 (5.31)	
30	148,488	104 (7.00)	
35	81,020	46 (5.68)	
Birth year			
2014	57,213	18 (3.15)	.028
2015	56,157	38 (6.77)	
2016	59,792	29 (4.85)	
2017	62,271	25 (4.01)	
2018	58,903	29 (4.92)	
2019	59,968	35 (5.84)	
2020	55,530	36 (6.48)	
2021	49,961	29 (5.8)	
2022	45,772	28 (6.12)	
2023	44,266	37 (8.36)	

**Figure 1.**
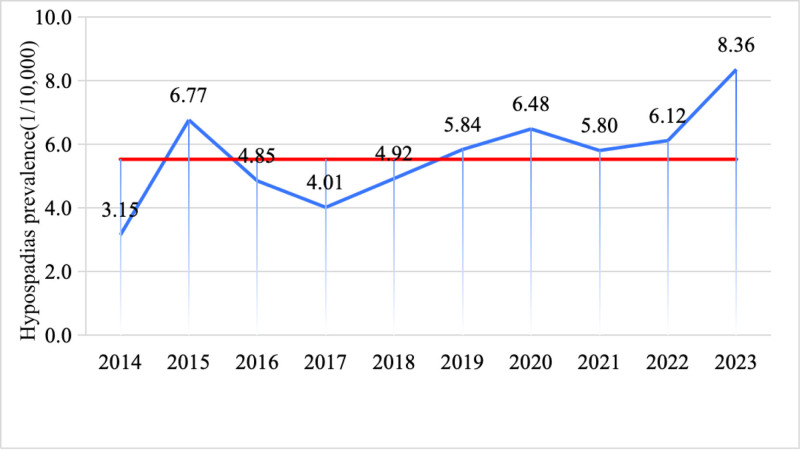
Hypospadias prevalence stratified by birth year.

According to the chi-square trend test, the overall prevalence of hypospadias from 2014 to 2023 demonstrated a significant increasing trend (χ²_trend = 8.48, *P* < .05). The lowest prevalence was recorded in 2014 at 3.15 per 10,000 births, while the highest prevalence was observed in 2023 at 8.36 per 10,000 births (Table 2 and Fig. 1).

A multivariate logistic regression analysis was conducted to evaluate the association between hypospadias and both residence and maternal age (Table 3). The results indicated significant relationships between hypospadias and maternal age (odds ratio: 1.82, 95% confidence interval: 1.02–1.26, *P* = .001).

**Table 3:** Multivariate logistic regression analysis for hypospadias by associated individual factors.

Variables	OR (95% CI)	*P*
Residence		
Urban	1.00	
Rural	0.80 (0.63–1.00)	.052
Age		
<20	1.33 (0.71–2.49)	.367
20	1.00	
25	1.38 (0.96–1.99)	.081
30	1.82 (1.27–2.62)	.001
35	1.48 (0.97–2.25)	.069

CI = confidence interval, OR = odds ratios.

## 4. Discussion

This study, based on data from the China Maternal and Child Health Surveillance System, reveals an increasing trend in the prevalence of hypospadias among newborns in Hainan from 2014 to 2023. A similar trend has been reported in Shanxi, Jiangsu, Zhejiang, and Hebei, China.^[15,16]^ The prevalence of neonatal hypospadias in Hainan was 5.53 per 10,000, with a total of 304 newborns diagnosed with hypospadias from 2014 to 2023. Our observed prevalence rates are notably higher than those reported in the Emilia Romagna region (2.26/1000),^[17]^ Italy (3.07/1000),^[18]^ and Hungary (2.25/1000),^[19]^ but lower than the international total (20.9/10,000).^[14]^ Additionally, it exceeds the total prevalence of hypospadias in Foz do Iguacu, Brazil (4.55/10,000),^[20]^ and parts of China (4.64/10,000).^[21]^ The reasons for our lower incidence rates remain unclear but they may be attributed to differences in study design, gestational age criteria, and diagnostic standards. The absence of uniform diagnostic criteria in many studies could also contribute to these variations.

Our study found no statistical difference in the prevalence of hypospadias among pregnant women living in rural areas compared to those in urban areas. However, some studies have indicated that the prevalence of neonatal hypospadias among pregnant women living in suburban and urban areas differs. Chen et al^[13]^ reported a lower prevalence of hypospadias in pregnant women living in suburbs compared to those in urban area and the counties (cities) under their jurisdiction. Similarly, Jin et al^[15]^ founded a higher prevalence of hypospadias in urban areas than in rural areas. But Xu et al^[16]^ reported a higher prevalence of hypospadias in rural areas than in urban areas. These discrepancies may be attributed to the varying criteria used to classify urban and rural areas during the study design. Hainan, being a relatively economically backward province, has ambiguous distinctions between urban and rural areas.

We identified an association between older maternal age, specifically within the 30 to 34 years age group, and an increased likelihood of Hypospadias. This finding is consistent with the research conducted by Fischl et al,^[22]^ who reported a correlation between advanced maternal age and the prevalence of hypospadias. Additionally, Ester et al^[23]^ confirmed that advanced maternal age may elevate the risk and predict the occurrence of these malformations.

The risk of hypospadias in the offspring of older pregnant women was found to be 3.86 times higher than that of younger pregnant women.^[13]^ Despite these findings, some studies have not confirmed an association between paternal age and hypospadias.^[24,25]^ Interestingly, the highest prevalence of hypospadias was observed among teenage mothers rather than older mothers.^[26]^ Consequently, we reached a similar conclusion. Our results showed that there was a significant difference in maternal age between the hypospadias case group and the control group. Further study of maternal age, its impact upon hypospadias, and its underlying genetic mechanisms warrant further investigation.

## 5. Strengths and limitations

Our study has several strengths. We conducted independent analyses of Hainan’s representative population-based surveillance data, which includes 549,833 birth records and 304 cases of hypospadias, providing a comprehensive overview of the prevalence and trends of hypospadias in Hainan. The prevalence of hypospadias was estimated using an active surveillance program that (1) minimizes information bias due to differences in surveillance methodologies, (2) serves as an effective approach to identifying hypospadias, and (3) reduces sampling bias due to the large population-based samples. However, our study must be interpreted in light of certain limitations. First, the data on hypospadias obtained in this study were not classified according to severity owing to the limited capacity of primary medical care. This may impact on the assessment of the prevalence and trends of hypospadias. Second, the study did not further investigate the correlation between various factors and neonatal hypospadias because due to the lack of comprehensive data on all newborns, such as birth weight, and gestational age. Finally, our study lacks detailed information on maternal lifestyle, environmental exposures, and genetic predispositions, which are crucial for understanding the etiology of hypospadias. Future research should address these limitations, incorporate more detailed data, and strive for improved quality and standardization of surveillance data to enhance our understanding of this condition.

## 6. Conclusions

In summary, our comprehensive study highlights an increasing prevalence of hypospadias among births in Hainan from 2014 to 2023. This prevalence exhibits notable variations associated with maternal age. The rising prevalence of hypospadias, along with its growing public health implications, underscores the urgent need for further research into the etiology, epidemiology, and clinical management of this condition within the Hainan population.

## Acknowledgments

The authors thank the obstetricians, pediatricians, pathologists, and other participants involved in the China Maternal and Child Health Surveillance System.

## Author contributions

**Conceptualization:** Weizhen Bu, Xiaohua Li, Xiaojing Hu, Yan Xuan, Zhenli Zhao.

**Data curation:** Weizhen Bu, Xiaohua Li.

**Formal analysis:** Weizhen Bu, Xiaohua Li.

**Investigation:** Weizhen Bu, Xiaohua Li.

**Methodology:** Weizhen Bu, Xiaohua Li.

**Software:** Weizhen Bu, Xiaohua Li.

**Writing—original draft:** Weizhen Bu, Xiaohua Li.

**Funding acquisition:** Xiaohua Li, Xiaojing Hu, Yan Xuan.

**Project administration:** Xiaohua Li, Xiaojing Hu, Yan Xuan, Zhenli Zhao.

**Writing—review & editing:** Xiaojing Hu, Yan Xuan, Zhenli Zhao.
